# Promoting Physical Activity among Workers: A Review of Literature and Future Directions on Developing Theory-Based Interventions

**DOI:** 10.3390/ijerph192013594

**Published:** 2022-10-20

**Authors:** Yan Sun, Yang Gao, Siyue Yu, Aiwei Wang, Xiaoting Ou, Dan Tao, Julien S. Baker

**Affiliations:** 1 Department of Sport, Physical Education and Health, Hong Kong Baptist University, Hong Kong 999077, China; 2 Centre for Health and Exercise Science Research, Hong Kong Baptist University, Hong Kong 999077, China; 3 JC School of Public Health and Primary Care, The Chinese University of Hong Kong, Hong Kong 999077, China; 4 College of Physical Education, Yangzhou University, Yangzhou 225012, China; 5 Department of Government and International Studies, Hong Kong Baptist University, Hong Kong 999077, China

**Keywords:** insufficient physical activity, working adults, theory-based intervention, intervention mapping, theories, behavior change techniques, delivery modes

## Abstract

Insufficient physical activity (PA) has been identified as a leading risk factor for premature and all-cause death, as well as non-communicable diseases. Employees, especially those with low occupational PA, are more vulnerable to physical inactivity, and studies in this population are scarce. However, employees may receive benefits for both health and work productivity from PA. Therefore, well-designed behavior change studies to promote PA in employees are urgently needed, especially during the COVID-19 pandemic. Literature was searched before 30 July 2022, and updated evidence was summarized. This review elaborates on the evidence related to insufficient PA and further provides an overview of theory-based interventions for promoting PA. Evidence indicates that intervention mapping (IM) was a useful tool to develop, implement, and evaluate behavior change interventions. Based on the IM framework, reviewing the theory- and evidence-based change methods and delivery modes, and further identifying the research gaps and limitations of existing interventions could provide promising suggestions and directions for development of well-founded interventions promoting PA among employees. The updated knowledge base for developing future interventions may boost efficacy and provide firm conclusions for researchers in this area.

## 1. Introduction

It has been well-documented that insufficient physical activity (PA) has been identified as a significant risk factor for early death and more than 25 chronic non-communicable diseases (NCDs), such as cardiovascular diseases (CVDs) (e.g., heart attacks, stroke), several cancers, respiratory diseases, and diabetes. Insufficient PA can also increase by 20 to 30% the risk of all-cause mortality compared to sufficiently active individuals [1,2], responsible for 7.2% and 7.6% of all deaths and CVDs deaths, respectively [3]. If people change their behavior to be more active worldwide, up to five million deaths per year could be averted [4]. Any duration of PA can accumulate these benefits. Additional benefits of physical fitness include, mental health, bone health, cognitive function, and quality of life [4]. Moreover, increasing PA will have profound impacts on health systems, economic development, environment, and society [5]. For example, working populations, who are the main force of labor productivity, will substantially reduce direct and indirect economic costs from health systems, improve worker productivity, and boost long-term economic development following PA increases [6].

Globally, 28% of adults were physically inactive before the pandemic of coronavirus disease (COVID-19). The World Health Organization (WHO) recommended levels of moderate to vigorous PA (MVPA) (i.e., 150–300 min of moderate-intensity of PA (MPA), or 75–150 min of vigorous-intensity of PA (VPA), or an equivalent combination of MPA and VPA per week) [1]. For working adults, 80% of this population spend one-third of their working day doing sedentary, desk-based tasks, this represents high exposure to insufficient PA and prolonged sitting time on workdays [7]. For example, a study reported that 50–60% of employees in Hong Kong had not engaged in any MVPA for at least 10 min a day [8]. This situation has been further exacerbated during the COVID-19 pandemic. Restriction measures, such as lockdown, social distancing, closures, self-quarantine, and work from home (WFH) hindered people from being active. Substantial evidence has consistently identified reduced levels of PA with concurrent increase in sedentary behavior in the general population [9,10]. For example, a cross-sectional study involving 13,503 adults (mean age: 39 ± 15 years) across 14 countries summarized that MPA and VPA decreased by 41.0% and 42.2%, respectively, compared to pre-pandemic values. Higher reduction in occupational PA (vs. leisure-time PA) was observed in working populations [11]. To avoid the amplification and continuation of this situation, there is an urgent need to promote PA, which would further reduce absenteeism and presenteeism among working populations.

After identifying the problem size of insufficient PA and its related outcomes, determinants (i.e., barriers and facilitators) of insufficient PA were also investigated to provide a solid foundation for selecting effective behavior change methods to promote PA [12]. Commonly identified barriers included “lack of knowledge”, “lack of time/competing demands on time”, “loss of interest/lack of motivation”, “lack of control” (e.g., lack of skills, facilities or equipment, space), “health-related problems”, “too harsh/lack of energy/feeling tired”, and some negative effects (e.g., fatigue, sweaty, too hot, embarrassed) [13,14] were all noted as barriers to PA. These notable barriers hampered the participation and promotion of MVPA among adults [15,16,17]. Some internal, external, and program-related facilitators of engaging in PA interventions were identified, such as “health-related needs and motivators”, “recommendation”, and “attractiveness of the program” [13].

Ample workplace intervention studies presented small effect sizes to improve PA and health [18], and no robust conclusions were produced from previous studies. This may be attributable to difficulties in identifying effective components of interventions, as a great heterogeneity of intervention design was observed, including intervention types and content, delivery modes, durations, PA measurements, quality of study design, etc. For example, a review included varied types of interventions to promote PA in employees and those with low-quality design, objectively measured PA, and delivered via the internet revealed better effectiveness [19]. In addition, another contributory factor may be that there was a lack of high-quality theory-based studies, which used valid theories and effective behavior change methods based on systematic frameworks to design, implement, and assess the interventions. Therefore, more well-designed studies are urgently needed to ameliorate existing research on determining the effectiveness of interventions in this population.

Previous literature indicated that theory-based interventions were considered to be more reliable and effective compared with non-theoretical ones [20,21,22]. Theory- and evidence-based approaches that focus on identifying effective behavioral change approaches and describing how these approaches are incorporated and developed have been widely used in feasible behavior change interventions [23]. Among these approaches, intervention mapping (IM), consisting of six steps, was a useful tool to design, implement, and evaluate behavior change interventions by synthesizing theories, evidence, and practice [12,24]. Based on the IM framework, effective methods to change behavior and appropriate modes to deliver these methods could be selected by reviewing the literature. Even if adopting the IM framework to develop a theory-based behavior change intervention is time-consuming, however, a high-quality study design could potentially boost the efficacy of the intervention and yield firm conclusions.

Previous research has demonstrated a notable gap of assessing efficacy of theory-based interventions to promote PA among working adults and identifying effective components of complex interventions. However, theory-based PA interventions with rigorous study design in this area are still lacking. More promising evidence from the literature regarding valid behavior change approaches is urgently needed for consideration in future research. Therefore, in this review, we provide an overview of theoretical foundations and existing interventions to promote PA in adults. We then integrate these findings to identify promising theories and methods, as well as outline research gaps for future studies. This current review provides health promotion planners multiple theoretical and experiential perspectives for the development of a well-designed theory-based behavior change intervention for the priority population.

## 2. Methods

Databases, including Scopus, PsycINFO, PubMed, Embase, Web of Science, and SportDiscus, were searched. Key search terms were combined by using Boolean operators AND/OR: including adult, worker, employee, workplace/worksite, physical activity (PA), exercise, health, cardiovascular disease (CVD), non-communicable disease (NCD), blood pressure, blood glucose, mental health, productivity, absenteeism, presenteeism, motivator, barrier, facilitator, qualitative, intervention mapping, theory-based intervention, and behavior change intervention. Search strategies were adjusted to fit different databases and only original studies and reviews published before 30 July 2022 in peer-reviewed journals in English were considered. Governmental reports and statistics (e.g., WHO) were also searched. In addition, reference lists of selected articles were considered and searched. Articles related to the three theoretical domains (i.e., IM, theories, BCTs) and existing PA interventions were extracted. The final pool consisted of 183 articles which were included in this review.

## 3. Results

### 3.1. Theoretical Foundations

There is an urgent call for behavior change studies to promote PA among working adults. Even if there is no consensus, theory-based interventions are widely considered to be more reliable and produce greater effects on influencing the determinants of behaviors and the mechanisms and pathways for changing behaviors than non-theoretical interventions [22,25]. Theory- and evidence-based behavior change interventions are suggested to identify “how” they can be developed conceptually (e.g., IM framework), “what” intervention contents are incorporated in methods (e.g., theoretical models), and “how” they can be delivered in practical context (e.g., modes of delivery, duration) [23]. However, there is a paucity of behavior change studies that integrate them to develop well-founded theory-based interventions [26,27].

#### 3.1.1. Intervention Mapping

IM is a planning framework that is based on a foundation of theoretical, empirical, and practical methodologies taking an ecological approach to understanding health problems and intervening at multiple levels (i.e., individual, interpersonal, organization, and community), because behavior occurs in complex ecological systems with distinct levels, linking different theories [12]. IM provides planners a guide to specify the problems and find solutions in two aspects: (1) evaluate a health problem, its behavioral and environmental factors, and determinants of behavioral and environmental factors; (2) identify precise and appropriate change methods to address determinants for the target behavioral and environmental factors [28,29]. For example, to develop an intervention to promote PA among employees, except for individual behavior, other environments, such as the physical environment of worksites, support from managers and colleagues, need to be considered for selecting valid behavior change methods [30,31,32].

**Figure 1:**
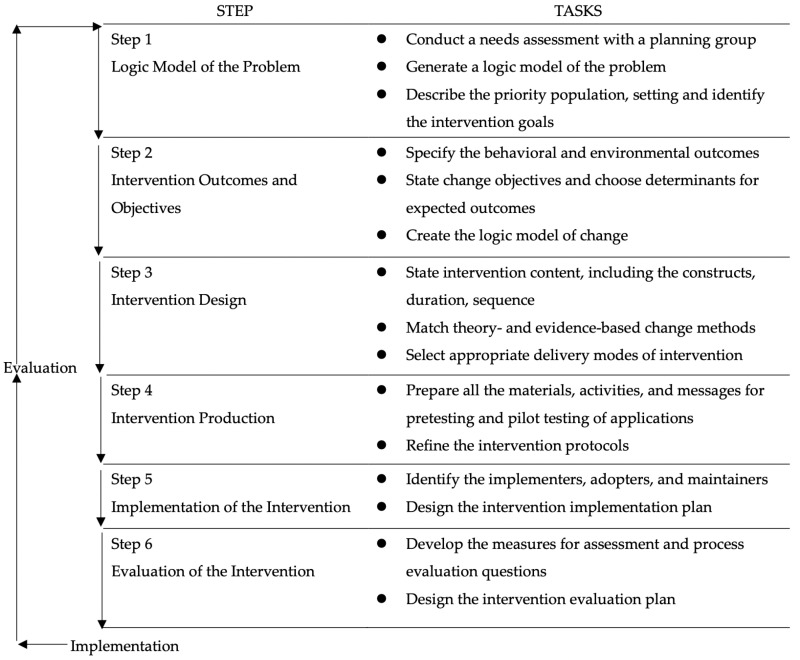
Intervention mapping steps and tasks (adapted with permission from Ref. [12]. 2016, Eldredge, L.K.B. *Planning Health Promotion Programs: An Intervention Mapping Approach*).

The IM framework consists of 6 steps. The first four steps are designed for development of the intervention, step 5 and step 6 are implementation and the evaluation plan. Each step comprises several tasks (Figure 1). Virtually, IM is an iterative process, which means that the completion of each previous step is a reference and guidance point for the next step. Intervention developers can move back and forth between tasks and steps to obtain more information and broader perspectives. Additionally, they can repeat or elaborate on steps as required. In addition, IM is also a cumulative process. If one of six steps is neglected, it may affect the validity of subsequent steps and the potential effectiveness of the entire intervention. After all steps are completed, a blueprint for design, implementation, and evaluation of the intervention is generated, containing intervention theories, experience, and practice.

The IM framework has been widely used in varied behavior change interventions. A systematic review has demonstrated that significant increases were observed in the uptake of disease prevention interventions [33]. In addition, IM has been used in a wide range of health promotion programs, targeting different populations (e.g., employees, older adults), in different contexts (e.g., workplaces, schools), and using different delivery modes (e.g., mHealth, web-based) [14,34,35,36,37,38]. Therefore, the IM approach is a promising tool to develop an intervention to improve PA and health in working adults, which may boost the effectiveness of the intervention and increase reliability of conclusions in this area.

#### 3.1.2. Theories of Behavior Change and Maintenance

Growing evidence has supported that well-designed and effective interventions were based on multiple theories to change and maintain behaviors [39,40]. Motivation and intentions were mostly targeted in many theories to change behaviors (e.g., the Theory of Planned Behavior) [41]. These theories suggested that lack of motivation was a major issue leading to non-engagement in healthy behaviors, so that increasing motivation would directly result in increased participation of behaviors. The observed shortage was that these theories focused on the relationship between intentions and behaviors without consideration of other decision-making constructs and processes in changing behaviors, indicating that motivation was necessary but insufficient to facilitate behavioral enactment [42,43]. Therefore, after intensions have been formed (motivational phase), the volitional phase theories suggested effective implementation strategies for behavioral enactment [44,45]. In addition, other “dual-phase” theories including the model of action phases and health action process approach contributed to strengthen the relationship between intensions and behavior [46,47].

Renewed “dual-process” theories indicated that behavior was influenced by motivational and volitional processes and identified that behavior might involve more implicit and non-conscious awareness [48,49,50,51]. “Dual-process” theories suggested two interacting processes for behavioral enactment: one was an imperative process that was determined by a rapid and low-consciousness process with low effort; the other one was a reflective process determined by a slower and deliberative process with considerable effort [52]. Behavior was initially controlled by a motivated transition to a deliberate approach, after which it occurred automatically through a habit-formation process [53]. Habit theories emphasized that behavior occurred by an impulsive, unconscious, and automatic process without deliberation. Mechanisms of habit formation might be formed by associating situations and behaviors and then repeating the behaviors that depended on the situation [54].

Based on the above evidence, multiple theories were recommended to change and maintain behavior; motivational and volitional phases in “dual-process” theories would increase engagement of behavior and habit theories would facilitate habit promotion. However, there is a scarcity of studies examining the effectiveness of interventions which adopted three theoretical processes (motivational, volitional, and habit-formation) for changing and developing behavioral habits.

#### 3.1.3. Behavior Change Techniques

Once the theories are identified, they will be linked with effective change methods or techniques and translated into practical applications [55]. No consensus has yet been reached on theory-informed interventions, which was attributed to insufficient details of intervention content and unclear descriptions of theory and practical strategy use in reporting complex intervention studies. This is challenging for researchers to identify effective intervention components, understand the links between theories and components, replicate them in future practical applications; and synthesize the evidence with intervention details to draw unanimous conclusions from systematic review studies [56]. However, these are essential in understanding the mechanisms of changing behaviors and translational processes, as well as increasing the potential effectiveness of interventions. Therefore, a formal system with standard definitions was recommended to specify the theories and intervention components. To achieve this goal, the UK Medical Research Council (MRC) guidance [57] advocated an advanced method (i.e., behavior change techniques (BCTs)) to effectively and efficiently describe the intervention content to resolve issues where there was no consensus.

Behavior change methods or techniques were identified and derived from content analysis from previous interventions [58,59,60,61]. Unique BCTs were identified as a set of terms to classify the techniques and used to specify the intervention content. Taxonomies, as classification systems, were developed as “active ingredients” for identifying specific intervention components and were applied to behavior change interventions (e.g., PA promotion and healthy eating) [40]. Recently, advanced, and comprehensive taxonomy contained more BCTs, thus needing a hierarchical structure, which would increase the coherence, usability, and application of the intervention [62]. BCTs taxonomy will produce several potential benefits: (1) the standardized taxonomies will increase the accuracy of replication of interventions; (2) clarifying intervention content through BCTs will promote high fidelity when delivering an effective intervention; (3) it is reliable for conducting systematic reviews to identify effective BCTs and synthesize the evidence to evaluate the effectiveness of complex interventions; (4) it will provide a definitely specified and detailed way to report interventions, and the comprehensive and structured list of BCTs makes the development of intervention simple and efficient; and (5) it is linked with theoretical constructs for understanding and investigating potential mechanisms of behavior change [40,63,64].

There have been numerous studies adopting BCTs to change health-related behaviors in distinct domains, including PA and healthy eating [40], smoking [65,66], and alcohol consumption [67]. Many systematic reviews specified the intervention components (e.g., delivery modes, intervention duration) of included studies and synthesized the evidence to evaluate the effectiveness of BCTs. For example, a systematic review investigating BCTs to improve PA in adults with overweight and obesity identified specific BCTs by delivery modes (i.e., face-to-face, and digital modes) and acknowledged the differences between them [68]. Another systematic review focused on the behavior change and maintenance, evaluating the effectiveness of PA interventions by using BCTs at post-intervention and at follow-up (i.e., 6 months or more) targeting healthy but physically inactive adults [69]. Based on the IM framework, effective BCTs will be selected and adopted targeting three distinct theoretical processes to increase PA (i.e., motivation, self-regulation, and habit formation).

#### 3.1.4. Delivery Mode of Interventions

A well-designed intervention is determined not only by identified themes, components, scope, and sequence, but also the delivery mode of the intervention. Multiple channels and vehicles could be selected according to preference and acceptability of intended target populations. Typically, a primary delivery method followed by a secondary method would reinforce the effectiveness of the behavior change interventions. Communication channels and vehicles have been widely used in previous interventions; channels included interpersonal, circulating print (or/and online), display print, radio, television, web-based, phones and smartphones, vehicles included community members, peer leaders, health care providers, online newspapers, posters, interviews, and text messaging [12]. These vehicles often target secondary populations, which could motivate behavior change objectives. Previous interventions mostly adopted an interpersonal communication channel, and vehicles might be community workers, teachers, or health care providers. They may act as facilitators to increase motivation by using methods and practical applications, for example, workshops, educational lectures, counselling, group discussions, and tutorials. In the real-world contexts, ecological interventions usually undertake a comprehensive approach at different levels, consisting of intervention means to change individual behaviors (e.g., workshop and counselling), organizational culture (e.g., organizational rules), and physical environment (e.g., posters and facilities) [70,71].

However, as the availability of digital technology has mushroomed, the proliferation of electronic health (eHealth) has been a new field delivered to serve health promotion, disease prevention and management programs. eHealth covers computer- and web-based tailored interventions, social media interventions, serious gaming interventions, and telephone and smartphone interventions [12]. Web-based intervention could be defined as a major self-guided online intervention program delivered by a website to create positive changes and/or improve/enhance knowledge, awareness, and understanding to help participants improve their physical health and mental health [72]. Thus, the development and dissemination of tailored web-based interventions seem to be more valuable and worthwhile [73]. There are some common advantages and disadvantages of web-based interventions. Advantages of web-based interventions have been demonstrated in previous studies. For example, web-based interventions could reach a large proportion of the population; effectively be applied in diverse populations; lower the cost; be personally tailored to suit both family and working circumstances, and participants could access flexibility in time and place for participation, and keep pace with themselves and ensure anonymity [73,74,75,76]. Web-based interventions have been widely applied in a multitude of practical applications (e.g., health promotion, clinical setting, health education, disease prevention), especially theory-informed interventions. For example, the comprehensive health enhancement support system (CHESS) adopted theoretical methods and models (e.g., problem solving, self-monitoring, and action planning) in varied domains, such as cancer, sexual assault, and alcohol consumption [77,78,79]. However, some limitations of web-based interventions have been identified in previous studies. For instance, web-based interventions usually indicated low participant engagement and retention rates due to a lack of interactions with participants and inability to involve verbal, aural and physical cues when delivering a web-based intervention [74]. Therefore, some face-to-face approaches (e.g., workshop and counselling) may have the potential to address the limitations of web-based interventions and boost the effectiveness of interventions.

More recently, blended interventions have emerged, which combined web-based and traditional face-to-face approaches and might be more effective than each individual approach in isolation [74]. Existing interventions have adopted this blended approach targeting patients for treatment, behavior change and self-management of diseases [74,80,81]. A meta-analysis included 11 studies involving overweight and obese adults (mean BMI of 32 kg/m^2^) with similar characteristics and found that blended interventions combining web-based and face-to-face could produce additional weight loss (MD: −1.48 kg, 95% CI: −2.52 to −0.43) compared with those that adopted face-to-face interventions alone. Whereas the face-to-face interventions were substituted with web-based interventions, significantly less weight loss was observed (MD: 1.47 kg, 95% CI: 0.13 to 2.81) [82]. Another systematic review of blended interventions to change behavior in patients with chronic somatic disorders indicated that the effectiveness of blended interventions was unclear and inconsistent for most outcome measures, which was probably due to the great heterogeneity of the type of intervention content, delivery modes, the form of presentation online, and integration of two separate modes [80]. Studies focusing on comparable outcomes to explore the superiority of blended interventions compared with stand-alone face-to-face or web-based interventions are scarce. Considering office workers who are equipped with computer and internet skills are the optimal target population, this blended approach is promising to promote PA and health, and more blended interventions are urgently needed to examine if they would yield larger effects than just web-based interventions.

### 3.2. Existing Interventions for Physical Activity among Adults

Although substantial evidence has acknowledged the benefits and efficacy of multiple types of interventions to promote PA and health, there was no consensus and robust conclusions from previous studies and the strength of the existing evidence was not substantial due to small effect sizes and statistically non-significant results [18,19,71,83]. With respect to workplace PA interventions, for example, a systematic review included three types of workplace PA interventions (i.e., PA or exercise interventions; counselling/coaching/support interventions; health promotion information interventions) with various delivery modes (e.g., face-to-face counselling or coaching, interviews). This study concluded that although some evidence identified efficacy of workplace PA interventions, no robust conclusions could be drawn due to the great heterogeneity of included studies, which stemmed primarily from intervention components, study designs, delivery modes, and intervention durations [18]. Another systematic review of workplace PA interventions further demonstrated the impact of heterogeneity on intervention effectiveness and found that studies adopted lower quality of study design, pedometers to measure PA, web-based modes, and targeted at multiple levels (i.e., social, environment) were more likely to report positive changes of PA [19].

In terms of web-based PA interventions, effectiveness would also be affected by the heterogeneity of interventions. A recent meta-analysis of web-based PA interventions reported positive effects on three types of PA levels (i.e., MVPA levels, step counts, minutes per week for walking), whereas heterogeneity regarding study quality, intervention duration, and variation of participants would influence the efficacy of the interventions [84]. In addition, engagement and retention of the participants should be focused on web-based PA interventions, because they would probably limit the effectiveness and implication of interventions. For example, an intervention study to promote PA from Australia evaluated the engagement and attrition rates for using the platform among 11,651 participants and reported that 50% and 25% of them kept using the platform after 30 days and 42 days, respectively. In addition, longer time of usage was related with lower engagement [73]. Similar results were observed in other web-based interventions [85,86,87]. Although most web-based PA interventions have reported positive effects, considering the low engagement and retention of participants, the effect size in the real world would be small [86]. Therefore, we need to investigate how people use web-based interventions; how to design and develop interventions producing sustainable effects; and how to attract participants regardless of whether they are highly motivated or not to promote their PA levels in real-world settings. There is also a need to establish both efficacy and effectiveness of interventions [88].

## 4. Discussion

Theory-based behavior change interventions are widely considered to be more reliable and effective than non-theoretical ones. IM is suggested as a guidance to develop, implement, and evaluate a well-designed intervention. Based on the IM framework, after the health problems, related risk behavior (i.e., insufficient PA), and its determinants are identified from the literature, then valid psychological theories, effective BCTs, and appropriate delivery modes are selected to effectively change the target behavior in intended populations.

Psychological theories can help us to understand the mechanisms and pathways of changing behaviors. To form a habitual behavior, evidence suggests that habit theories (habit-formation process) combined with updated “dual-process” (motivational and volitional) might increase the automaticity of behavior and demonstrated the maintenance effect of interventions. Therefore, future theory-informed interventions to promote PA and health could be directed towards combining these three theories to change and maintain the behavior. Linking effective BCTs and theoretical constructs with appropriate theories could bridge the gap between theory and practice. Updated BCTs taxonomy targeted distinct theories could help to identify the effective components of complex interventions, improve the efficiency of development of interventions, provide reliable conclusions of effectiveness from the literature, and increase fidelity and replication in future research. This will boost the potential effectiveness of interventions and yield firm conclusions.

The capability of both workplace and web-based PA interventions was affirmed from the literature, however, effect sizes were small, and results were inconclusive. Great heterogeneity across interventions still exists. There is an urgent need to identify the particular intervention elements, that are associated with increased efficacy of intervention outcomes and maintenance effects of interventions. Future research directions should focus on interventions with a high-quality study design, clear and detailed intervention components, long-term durations, as well as attractive programs (e.g., preferences of participants regarding content, features, or the delivery mode) for the target population with larger sample sizes [89].

## 5. Conclusions

Theory-based interventions which are delivered using a blended approach are promising and feasible to promote PA and health among adults, especially working population. Future studies with rigorous experimental methods, long-term follow-ups, well-designed intervention protocols (e.g., content, theories, framework, BCTs), appropriate modes and formats of delivery are suggested and expected to promote efficacy of interventions and draw well-founded conclusions in future research.

Evidence extracted from the literature has identified research gaps in existing interventions and provided suggestions and directions for the development of an effective and reliable intervention for the priority population. If the effectiveness of this promising intervention methodology is confirmed, PA levels, health, and work productivity may be improved. The results will add knowledge to the area of identifying effective components of theory-based interventions and assessing the effectiveness of a blended approach.
